# Fracture behaviors of pre-cracked monolayer molybdenum disulfide: A molecular dynamics study

**DOI:** 10.3762/bjnano.7.132

**Published:** 2016-10-07

**Authors:** Qi-lin Xiong, Zhen-huan Li, Xiao-geng Tian

**Affiliations:** 1 Department of Mechanics, Huazhong University of Science & Technology, 1037 Luoyu Road, Wuhan 430074, China; 2 Hubei Key Laboratory of Engineering Structural Analysis and Safety Assessment, 1037 Luoyu Road, 430074, Wuhan, China; 3 State Key Laboratory of Strength and Vibration, Xi’an Jiaotong University, Xi’an 710049, China

**Keywords:** crack propagation, fracture strength, molecular dynamics simulation, monolayer molybdenum disulfide, pre-existing crack

## Abstract

The fracture strength and crack propagation of monolayer molybdenum disulfide (MoS_2_) sheets with various pre-existing cracks are investigated using molecular dynamics simulation (MDS). The uniaxial tensions of pre-cracked monolayer MoS_2_ sheets with different crack tips, different locations of crack, different crack lengths and angled cracks are simulated and studied. The results show that the configuration of crack tip can influence significantly the fracture behaviors of monolayer MoS_2_ sheets while the location of crack does not influence the fracture strength. With the increase of crack length, the fracture strength of monolayer MoS_2_ sheets reduces almost linearly, and the fracture of monolayer MoS_2_ sheets is transformed from almost brittle to ductile. By making comparison between the MDS results and the predictions of continuum fracture mechanics theories, including Inglis' model, Griffith's model with and without finite size effect, it is found that MDS results agree well with the predictions of Griffith's model with finite size effect, differ from the predictions of Inglis' model and Griffith's model without finite size effect. Finally, the MDS results of monolayer MoS_2_ sheets with different angled crack are also analyzed based on the continuum fracture mechanics model.

## Introduction

It is well known that the existence of crack in the real material is inevitable [1]. However, the unavoidable cracks have a significant influence on the mechanical strength of materials and even cause catastrophic events, thus the investigation of the fracture behavior of materials with pre-existing cracks is of great significance in engineering application. Metal dichalcogenides such as MoS_2_ and WS_2_ are well known for their interesting catalytic, photovoltaic, and lubricant properties [2–4] making them of interest for a variety of potential applications including scanning probe tips, high strength nanocomposites and nanostructures, mechanical devices, and electronics [5–6]. In order to develop MoS_2_-based nanocomposites and nanostructures with improved their performance, further fundamental and applied research on MoS_2_ is essential [7]. Since the desired applications including high strength nanocomposites and nanostructures require MoS_2_ to undergo the mechanical loadings such as tension, a thorough understanding of the mechanical behavior of MoS_2_ under mechanical loading is essential for its applications in nanocomposites and nanostructures.

The mechanical properties of the defect-free MoS_2_ sheets have been investigated by many researchers using different methods. Cooper et al. calculated the nonlinear elastic response of two-dimensional MoS_2_ with first-principles density functional theory (DFT) method [8]. Castellanos-Gomez et al. [9] performed bending test experiments by using the tip of an atomic force microscope (AFM) and measured the elastic properties of freely suspended multi-layered MoS_2_ nanosheets (5 to 25 layers). Bertolazzi et al. [10] reported on measurements of the stiffness and breaking strength of monolayer MoS_2_, found the effective Young's modulus of monolayer MoS_2_ is 270 ± 100 GPa and breaking occurs at an effective strain between 6 and 11% with the average breaking strength of 23 GPa.

Additionally, compared with the first-principles DFT and experimental approaches, MDS method has advantages in the computational cost and catching details [11–14]. Jiang et al. [15] presented a parameterization of the Stillinger–Weber (SW) potential to describe the interatomic interactions within single-layer MoS_2_ (SLMoS_2_). And based on this potential, they studied chirality, size, and strain effects on the Young’s modulus and the thermal conductivity of defect-free SLMoS_2_ by using classical MDS. Dang et al. [16] used MDS with a reactive empirical bond order potential to study the mechanical deformation and failure of monolayer molybdenum disulfide under uniaxial and multiaxial tension. Zhao et al. [17] investigated the influence of temperature on mechanical properties of single-layer MoS_2_ by adopting MD nanoindentation simulation and found that with the increase of temperature (4.2 K to 500 K), the Young’s moduli, fracture stress and strain of MoS_2_ decrease and they also studied the tension-induced phase transition of single-layer MoS_2_ at low temperatures by applying MDS [18].

Although there have been many studies on the mechanical properties of the defect-free MoS_2_ sheets, very few report about the investigation of the fracture behavior of the MoS_2_ sheets with pre-existing cracks can be found. Recently, Wang et al. [19] studied the fracture toughness and crack propagation path of monolayer MoS_2_ sheets with edge crack under mixed modes I and II loading by using MDS. In the present work, we focus on investigating the effect of crack tip configuration, crack location, crack length and angled crack on the fracture strength and crack propagation of pre-cracked monolayer MoS_2_ sheets by using MDS. By making comparison between the MD results and the predictions of continuum fracture mechanics theories, including Inglis' model and Griffith's model with and without finite size effect, it is found that MDS results agree well with the predictions of Griffith's model with finite size effect, differ from the predictions of Inglis' model and Griffith's model without finite size effect. Finally, the MDS results of monolayer MoS_2_ sheets with different angled crack are also analyzed based on the continuum fracture mechanics model.

## Process of molecular dynamics modeling

**Figure 1:**
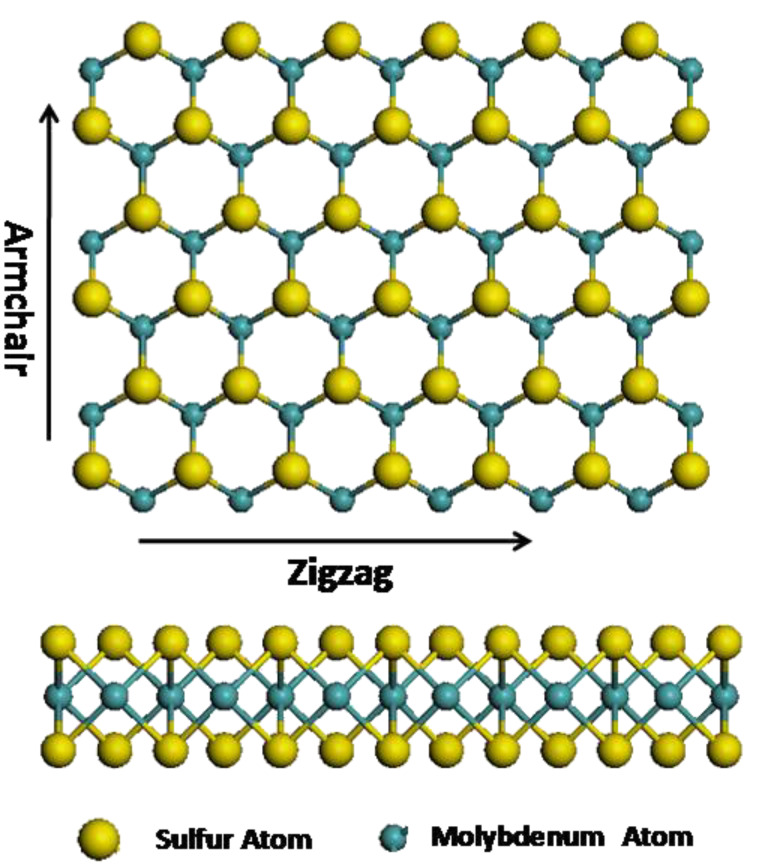
The atomic structure of a defect-free MoS_2_ sheet.

To study the fracture behaviors of MoS_2_ sheets with different pre-existing cracks, the MD model of a defect-free MoS_2_ sheet composed of 31680 atoms (10560 Mo and 21120 S atoms) was built, with the spatial dimension of 299.5 Å × 297.6 Å as shown in Figure 1 (only part of the model is displayed), unless otherwise specified, this model size will be used in most of the simulations.

The SW potential [15] is adopted to describe the interaction between molybdenum and sulfur atoms in the MoS_2_ sheets due to its good agreement with the experimental data and its successful usage in the previous studies [15–17]. The MD simulation technique described in the existing study [11] is applied in the present work and the periodic boundary condition is applied in all directions of model to remove the effect of finite size and the time steps selected are 1.0 fs for all the MD simulations in the present study. Before the displacement load is applied, the MD systems are equilibrated to 1 K to reduce the thermal fluctuation effect [20–22] and traction free by relaxing the system for 50 000 steps with the use of Nose–Hoover style thermostat and barostat (NPT) [23]. And then the system is further regarded as microcanonical ensemble (NVE) and equilibrated for 20 000 time steps. Since the difference is slight between the results at the strain rate of 10^8^ s^−1^ and 10^10^ s^−1^, the constant strain rate of 10^9^ s^−1^ is applied in every uniaxial tension simulation of present work. Additionally, to calculate the stresses of sheets [11], the thickness of monolayer MoS_2_ sheets is taken as 0.61 nm, which is close to the spatial distance between layers of MoS_2_ sheets.

## Results and Discussion

### Uniaxial tension of defect-free MoS_2_ sheet

**Figure 2:**
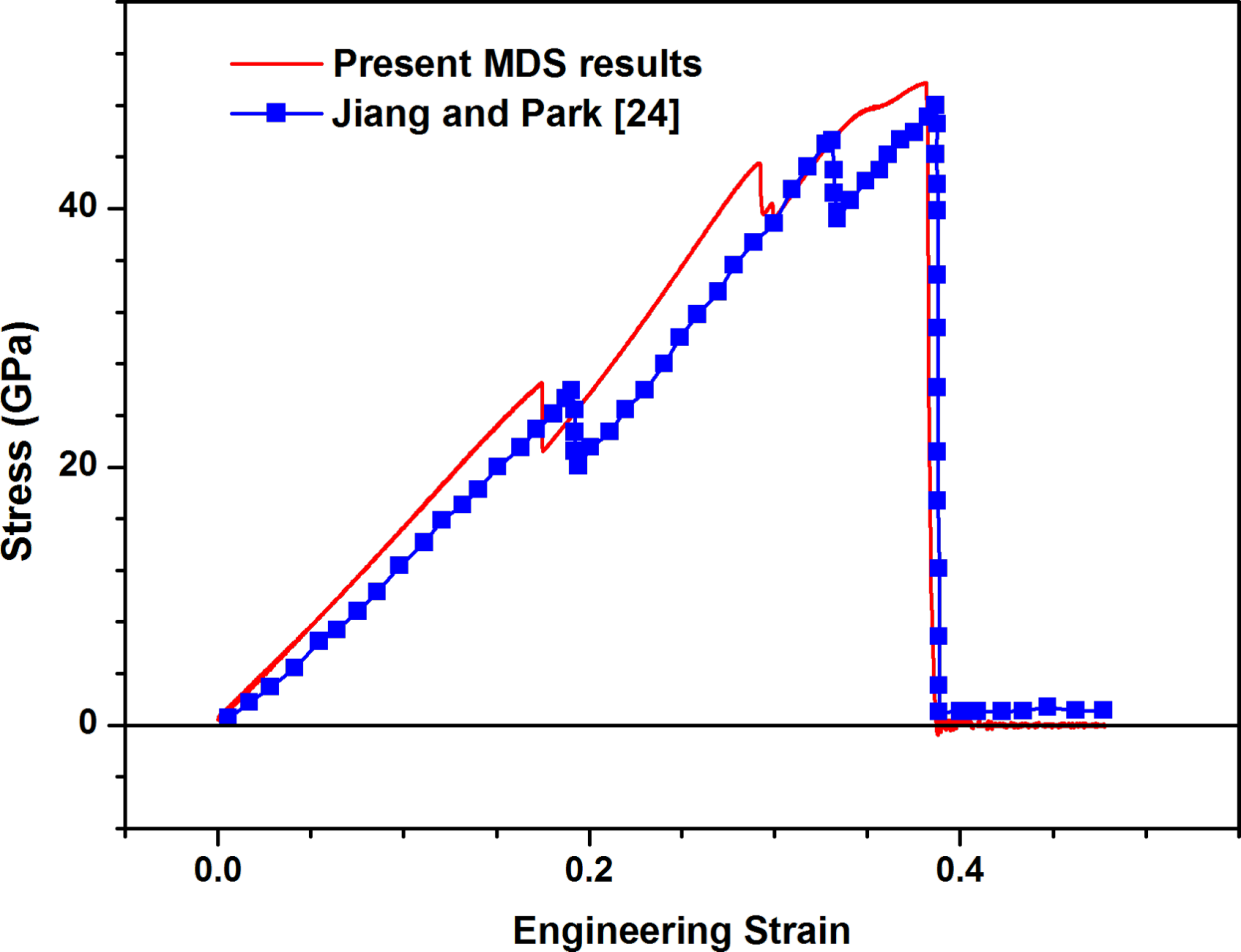
Comparisons of uniaxial tensile stress–strain curve along armchair direction of defect-free MoS_2_ sheet between the present study and the existing literature [24].

To verify the MDS method employed in the present work, the uniaxial tension of a MoS_2_ sheet in the existing literature [24] is simulated. Figure 2 shows the comparison between the uniaxial tensile stress–strain curves of defect-free MoS_2_ sheet in the present study and the results of Jiang and Park [24] for armchair direction, the stress–strain curve in the present study agrees with the result of Jiang and Park [24], demonstrating the effectiveness of the present MDS method. The discrepancy in the numerical value can be attributed to the numerical calculation error and the difference of modeling details. For the armchair direction, the Young's modulus is estimated at 150 GPa by calculating the slope of linear part in the whole curve, which is comparable with the experimental data of about 270 ± 100 GPa from Bertolazzi et al. [10]. However, the fracture strength of about 50 GPa and the fracture strain of about 38% in the present MDS significantly distinguish from the results obtained using experimental measurement by Bertolazzi et al. [10], the present numerical simulation results are far greater than the experimental results. The significant discrepancy can be attributed to the inevitable defect in the experimental specimen and the difference of load types (uniaxial tension for the current simulation and multiaxial tension for the experiments), and it is also the motivation to carry out the present work.

**Figure 3:**
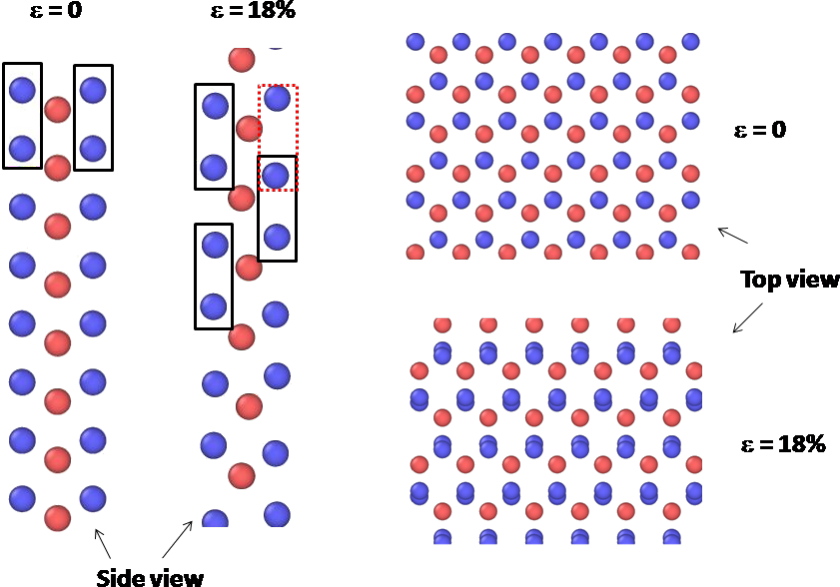
The top and side views of atomic structures under different uniaxial tension strains along the armchair direction of defect-free MoS_2_ sheet.

As described in the previous work of Jiang and Park [24], we also have observed the structure transition of MoS_2_ sheet in the process of tension simulation. The top and side views of atomic structures under different uniaxial tension strains are shown in Figure 3. From Figure 3, the structure consisting of molybdenum and sulfur atoms varies suddenly at the engineering strain of about 18%. Please see Supporting Information File 1 and Supporting Information File 2 for a clear observation of the structure transition of MoS_2_ sheet.

### The effect of crack tip

**Figure 4:**
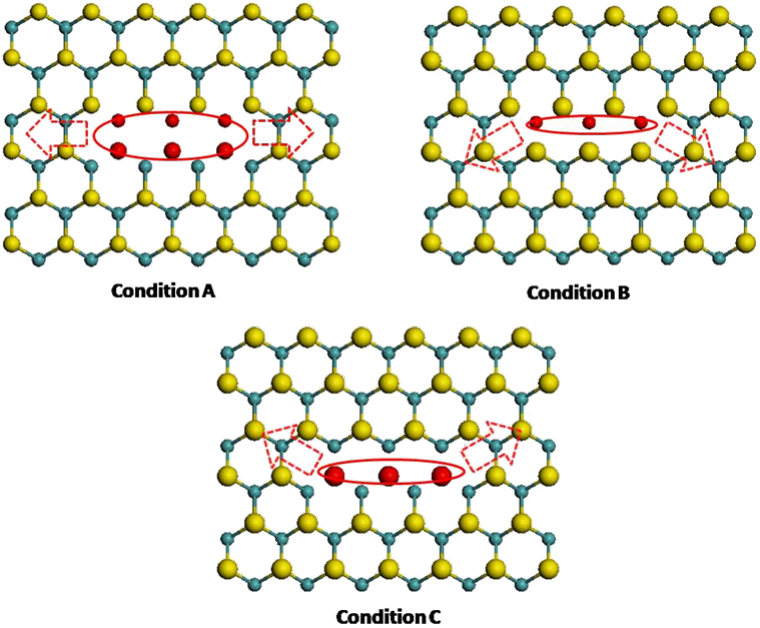
Three types of crack tip configuration with 1nm length: condition A, a Mo atom and two S atoms at the crack tip; condition B, four S atoms at the crack tip; condition C, two Mo atoms at the crack tip.

To investigate the effect of crack tip on the fracture behaviors of pre-cracked monolayer MoS_2_ sheets, three types of crack tip configuration with equal crack length of 1 nm are constructed as shown in Figure 4, such as a Mo atom and two S atoms at the crack tip for the condition A, four S atoms at the crack tip for the condition B and two Mo atoms at the crack tip for the condition C.

**Figure 5:**
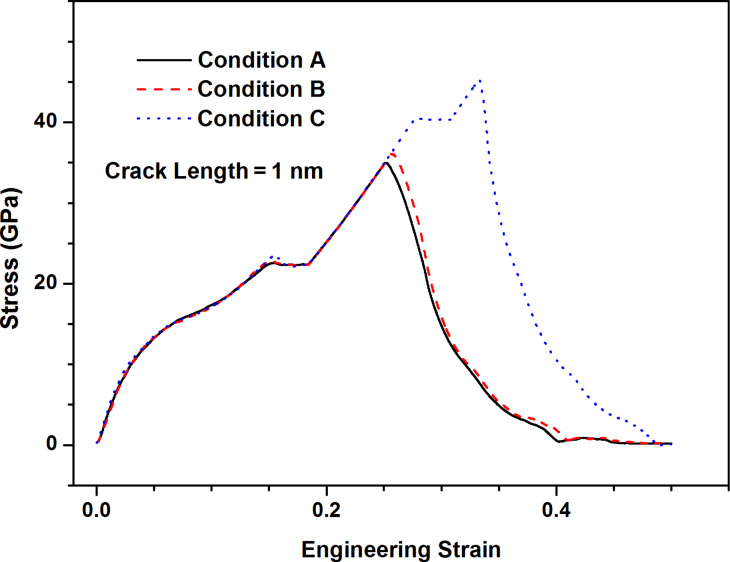
The stress–strain curves along armchair direction of MoS_2_ sheet under three crack tip types: condition A; condition B; condition C.

Figure 5 presents the stress–strain curves along armchair direction of MoS_2_ sheet with 1 nm crack under three types of crack tip configuration. From Figure 5, the fracture strength of the condition B is slightly higher than that of the condition A while the fracture strength of the condition C is much higher than that of the condition A, which indicates the configuration of crack tip has a significant influence on the fracture strength. It is worth noting that there always exist kink angles in the crack tips of the condition B and the condition C, the kink angles are 30 degree and −30 degree for the condition B and the condition C, respectively. According to the continuum fracture mechanics model [1], the fracture strength of pre-cracked MoS_2_ sheet is

[1]
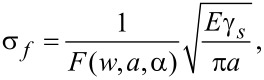


where *E* is Young’s modulus, γ*_s_* is the surface energy and *F*(*w*,*a*,*a*) is a function depending on the geometry parameters as defined in Equation 2.

[2]
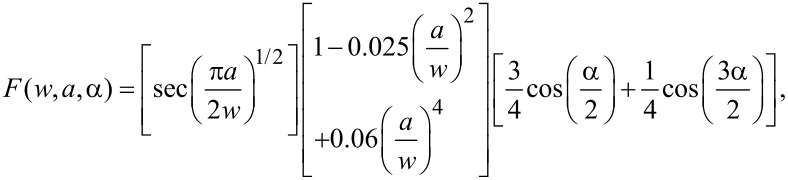


In Equation 2 *a* denotes a half of crack length, *w* is a half of MoS_2_ sheet width, and α is the kink angle of crack tip.

**Figure 6:**
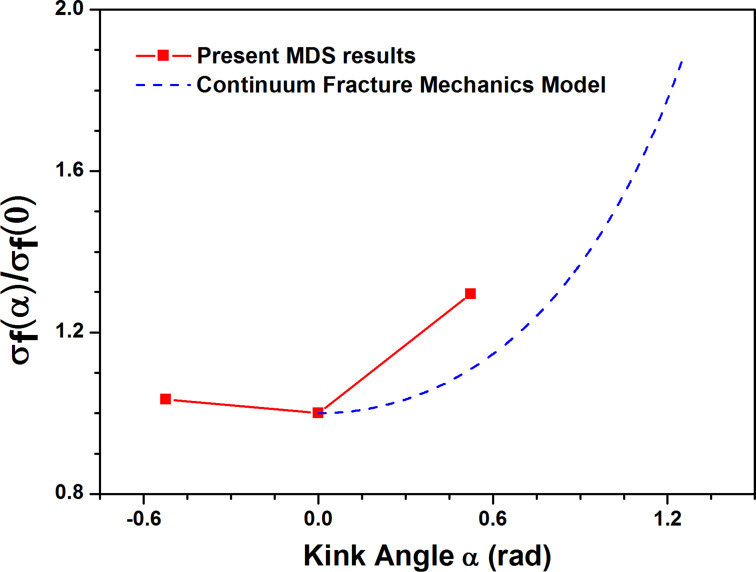
Comparison of fracture strength of MoS_2_ sheet with the crack of 1 nm length between MDS results and continuum fracture mechanics model versus the different kink angle α, i.e., different crack tips.

For the condition A, the kink angle of crack tip is considered to be 0 degree. Figure 6 shows the comparison of fracture strength of MoS_2_ sheet with the crack of 1 nm length between MDS results and continuum fracture mechanics model. The trend of MDS results agrees with the prediction of continuum fracture mechanics model although there are numerical differences between them, intimating the kink angle of crack tip has influence on the fracture strength.

The fracture strengths of the condition B and condition C should be equal according to the prediction of continuum fracture mechanics model (Equation 1). However, the MDS results of both conditions show great difference. This shows that the effect of crack tip is not only related to the kink angle of crack tip, but also other factors.

There are four S atoms at the crack tip and the S–S bonds are formed for the condition B while the Mo–Mo bond is built for the condition C, the S–S bond is much weaker than the Mo–Mo bond according to the description of SW potential [15]. Additionally, the crack always starts from the location which has the weak bond [11]. Thus, the fracture strength of condition C should be higher than that of condition B as shown in Figure 6.

**Figure 7:**
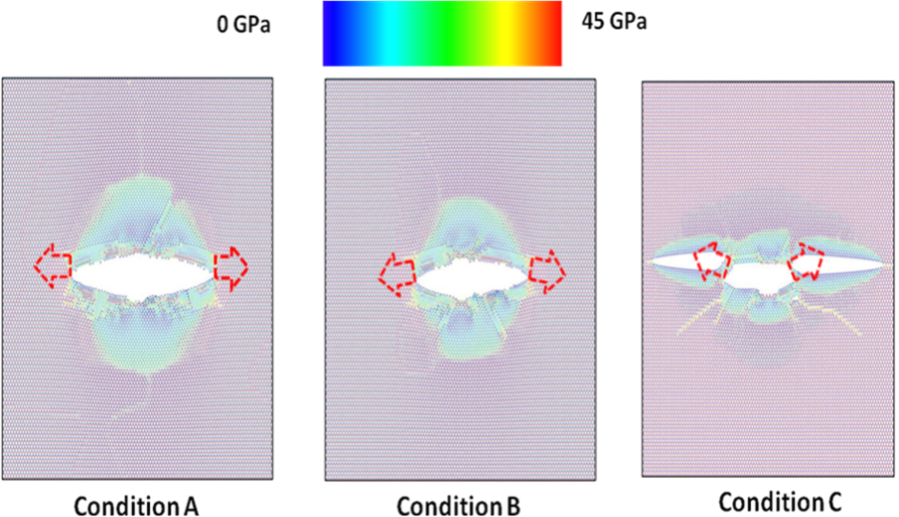
The von Mises stress distribution after crack extension for three crack tip types.

**Figure 8:**
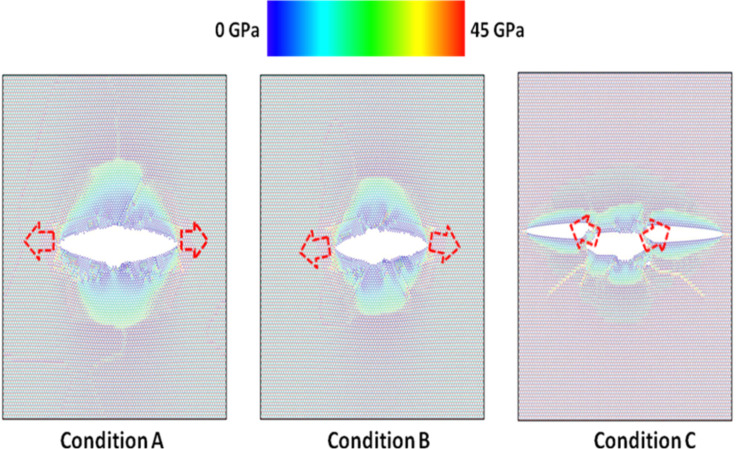
The maximum principal stress distribution after crack extension for three crack tip types.

In order to understand more clearly the effect of crack tip on the fracture behaviors of pre-cracked monolayer MoS_2_ sheets, the von Mises stress distribution (usually resulting in ductile fracture) and the maximum principal stress distribution (usually resulting in brittle fracture) at the atomic structure after crack extension for three crack tip types are presented in Figure 7 and Figure 8, respectively. From Figure 7 and Figure 8, it can be clearly seen that the directions of crack propagation are different under three crack tip types, and the crack tends to propagate along the kink angle of crack tip. The results further shows the crack tip has a significant influence on the fracture behaviors of pre-cracked monolayer MoS_2_ sheets, which agree with the prediction of continuum fracture mechanics model considering the kink angle of crack tip [1].

### The effect of model size and crack location

**Figure 9:**
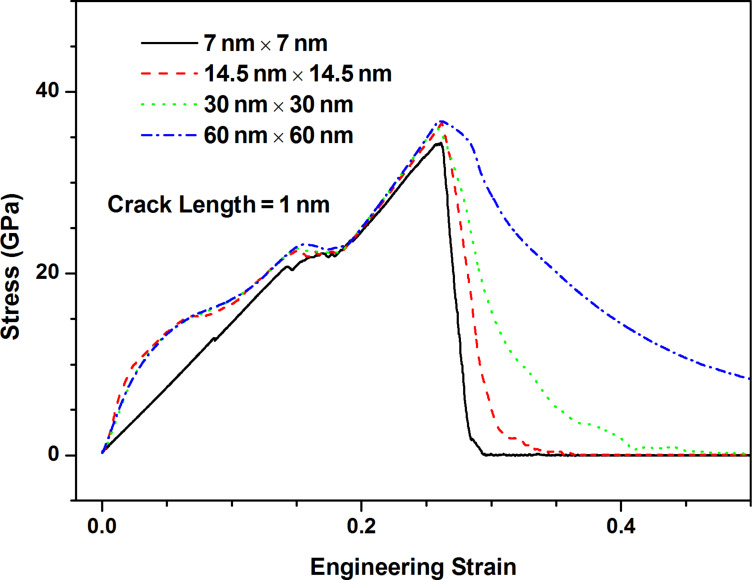
The stress–strain curves along armchair direction of MoS_2_ sheet with the crack of 1 nm length under four sizes: 7 nm × 7 nm; 14.5 nm × 14.5 nm; 30 nm × 30 nm; 60 nm × 60 nm.

**Figure 10:**
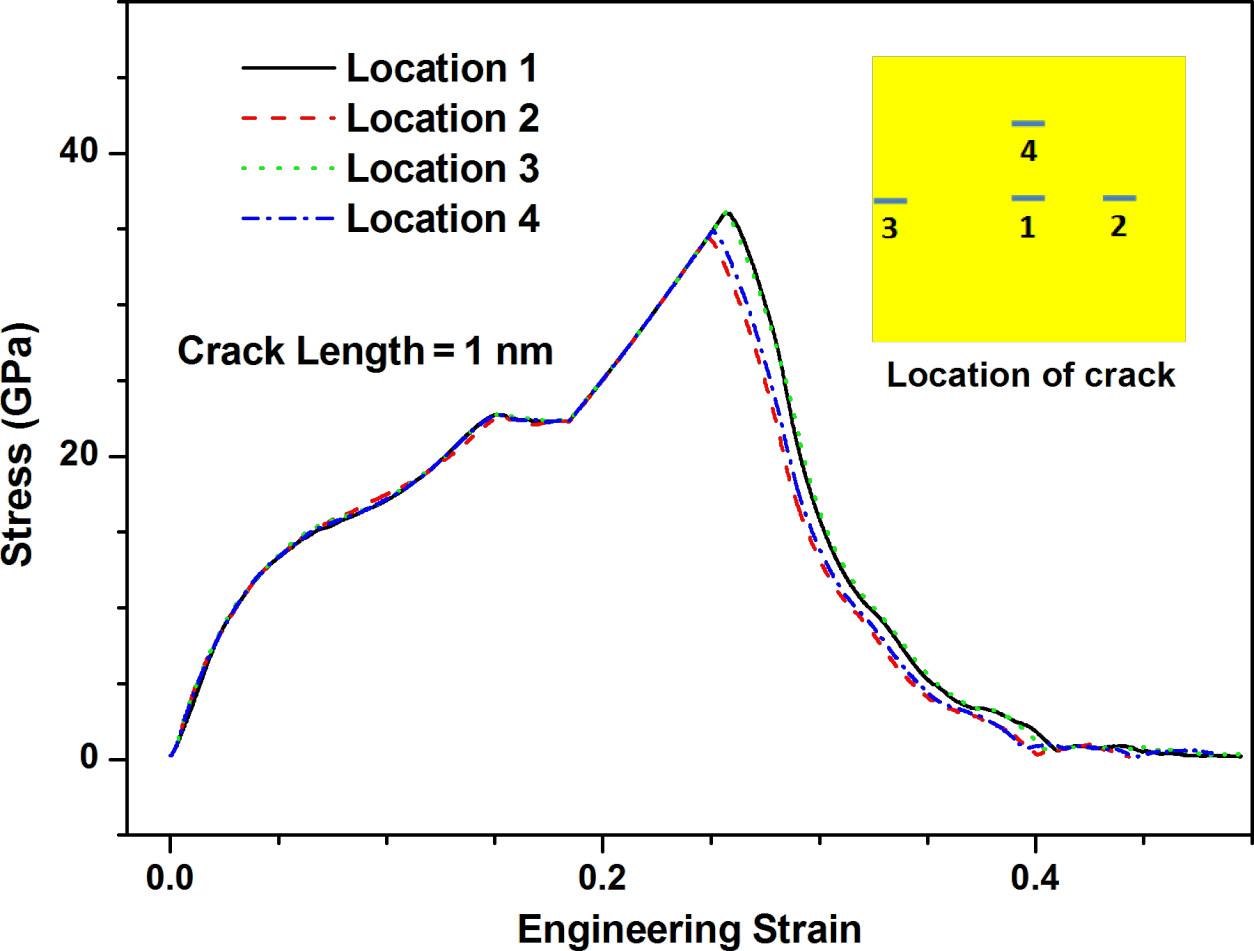
The stress–strain curves along armchair direction of MoS_2_ sheet with the crack of 1 nm length for different crack locations.

Figure 9 shows the stress–strain curves along armchair direction of MoS_2_ sheet with the crack of 1 nm length for four model sizes considered. With the increase of model size, the fracture strength increases very slowly and the variation of value is very small. The difference between fracture strength estimation from the Griffith model with considering finite size effect (Equation 1) for four model sizes is very slight (about 5%), thus the observation of fracture strength shown in Figure 9 is understandable. However, fracture behavior is gradually transformed from brittle to ductile with the increase of model size. The potential reason is that the crack propagation is at the finite speed, and thus the larger the model size is, the longer the fracture process becomes. From Figure 10, the location of crack has not any influence on the fracture behavior of pre-cracked MoS_2_ sheets due to the usage of periodic boundary conditions.

### The effect of crack length

**Figure 11:**
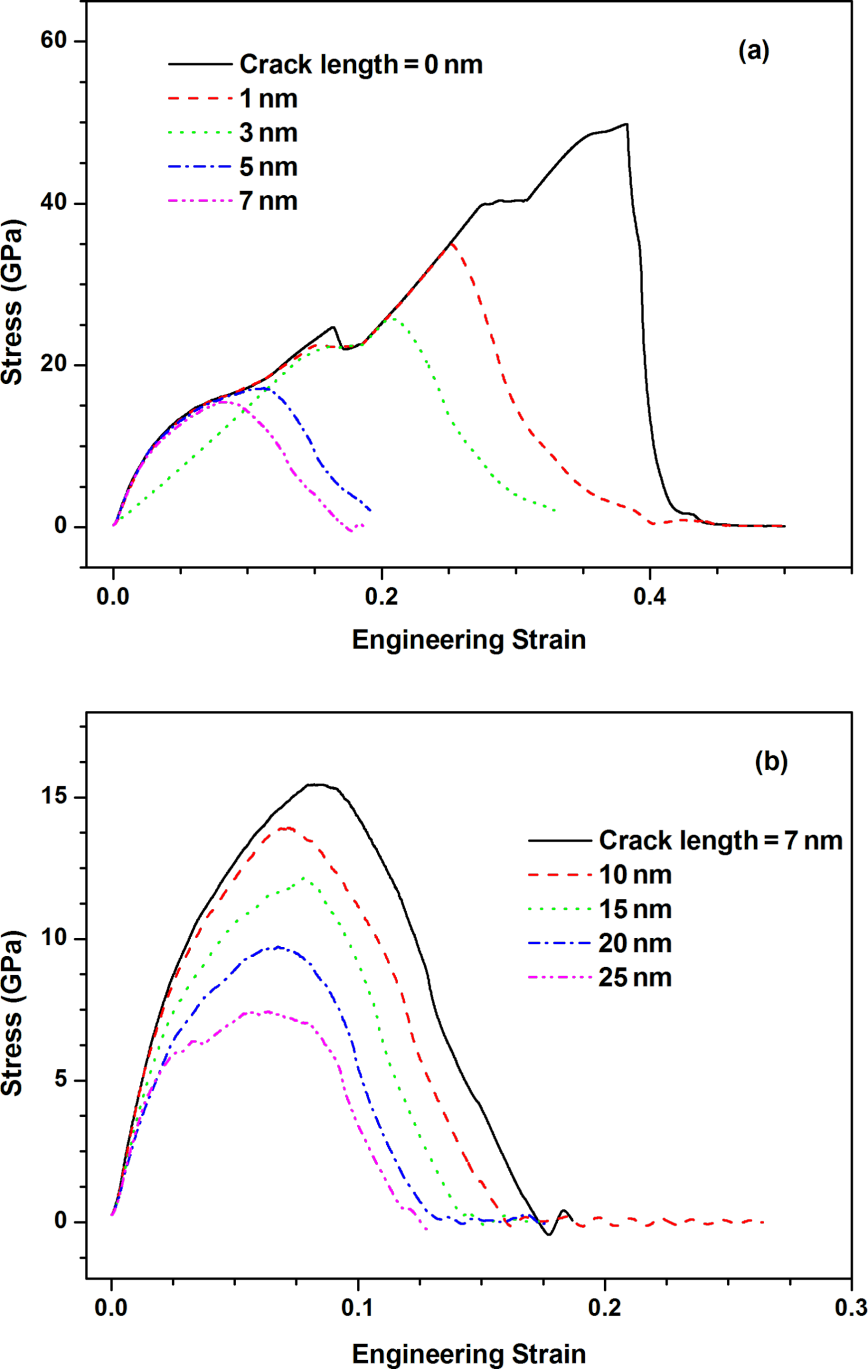
The stress–strain curves along armchair direction of pre-cracked MoS_2_ sheet with different crack lengths: (a) 0–7 nm; (b) 7–25 nm.

**Figure 12:**
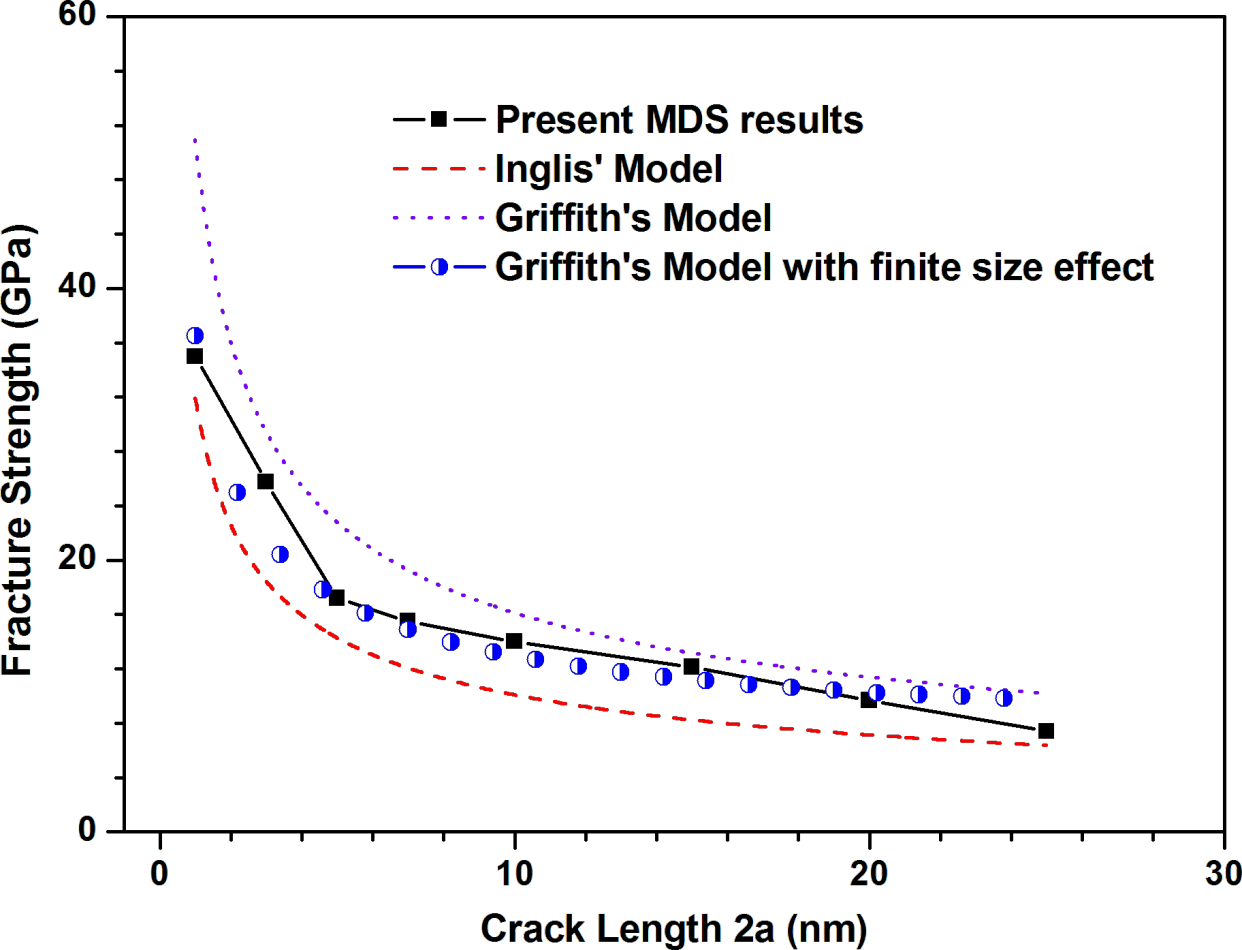
Comparison of fracture strength of MoS_2_ sheet between MDS results and three continuum fracture mechanics models versus different crack lengths.

Figure 11 shows the stress–strain curves along armchair direction of pre-cracked MoS_2_ sheet with different crack lengths. With the increase of crack length, the fracture strength of pre-cracked MoS_2_ sheet reduces almost bi-linearly as shown in Figure 12. In order to better understand the effect of crack length on the fracture behaviors of pre-cracked MoS_2_ sheet, two continuum fracture mechanics models, i.e., Inglis' model [25] and Griffith's model [26] as expressed by Equation 3 and Equation 4 without finite size effect, are used to estimate the fracture stresses of pre-cracked MoS_2_ sheets with different crack lengths.

[3]
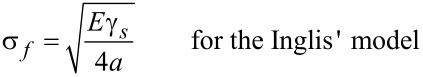


[4]
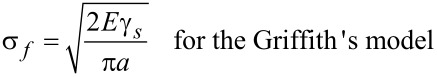


The Equation 1 in the preceding section is Griffith's model with finite size effect. The surface energy can be estimated by the difference of potential energy of system before and after fracture. However, due to the significant difference between the fracture surface of different simulations, the surface energy varies dramatically (0.5–10 J/m^2^). With Young’s modulus calculated above and taking the surface energy of MoS_2_ sheet to be 5 J/m^2^ for calculating, the results obtained from three continuum fracture mechanics models are compared with the present MDS results, as shown in Figure 12.

The present MDS result for the fracture strength of MoS_2_ sheet agrees well with the prediction of Griffith’s model with finite size effect when the crack length is less than 20 nm. However, compared with the present MDS result, the results predicted by Inglis' model are always lower, while the results of Griffith’s model are always higher when the crack length is less than 20 nm. This demonstrates that it is very necessary to take account of the finite size effect in estimating the fracture stress of pre-cracked MoS_2_ sheet theoretically. When the crack length is beyond 20 nm which is close to the model size, the predictions of Griffith’s model with and without finite size effect seem no difference and the Griffith’s model with finite size effect also fails at this time. Therefore, when predicting the fracture strength of pre-cracked MoS_2_ sheet theoretically, we need to consider the comparability between crack length and model size. Only when the crack length is less than the model size, the theoretical prediction is reliable and credible. A possible reason is that when the crack length is close to the model size, interaction between two cracks due to the considered periodic boundary condition becomes strong.

Additionally, it is a very interesting phenomenon that the brittle fracture of defect-free MoS_2_ sheet is changed into ductile fracture of pre-cracked MoS_2_ sheet when a crack exists in the MoS_2_ sheet. To reveal the potential reason, the speed of crack propagation estimated based on continuum fracture mechanics model [1] is given as follows:

[5]
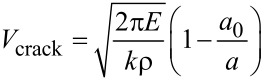


where ρ is density and *k* is a material constant, *a*_0_ is the initial crack length, *a* is the present crack length. From Equation 5, the speed of crack propagation descends with the increase of initial crack length under the condition that other parameters are invariable. The lower the speed of crack propagation is, the longer the fracture process becomes for the equal model size. Thus, the phenomenon shown in Figure 11 is understandable.

### The effect of crack angle

According to the continuum fracture mechanics theory [1], when a crack is not perpendicular to the direction of loading and it is oriented at an angle β, we can introduce an effective Mode I crack. For the angled crack in the present work, the equivalent crack length is shown in Equation 6 [1].

[6]
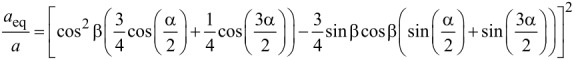


For the crack tip of the condition A, the kink angle α is 0 degree, thus, the Equation 6 can be reduced as

[7]
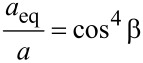


**Figure 13:**
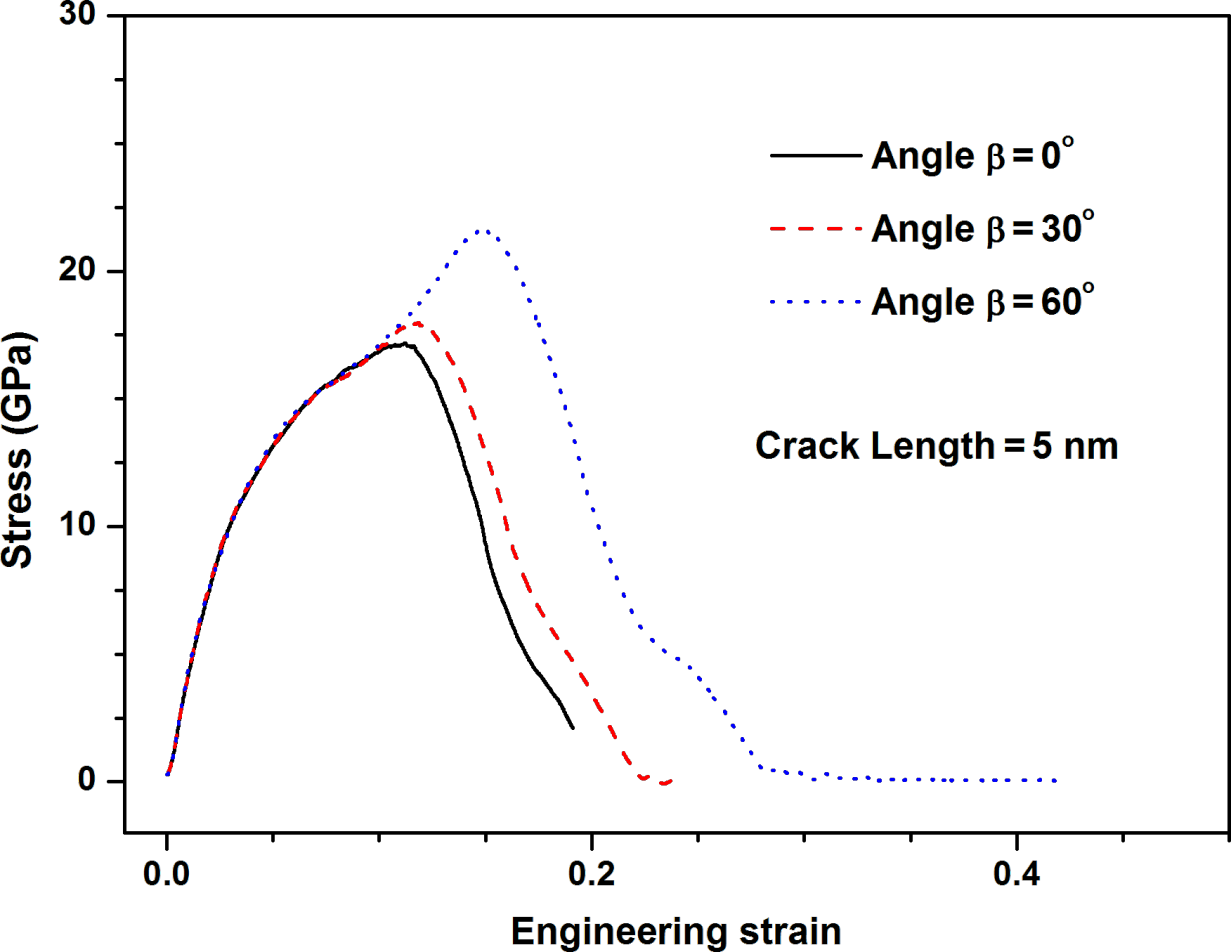
The stress–strain curves along armchair direction of MoS_2_ sheet with different crack angles: β = 0°; β = 30°; β = 60°.

**Figure 14:**
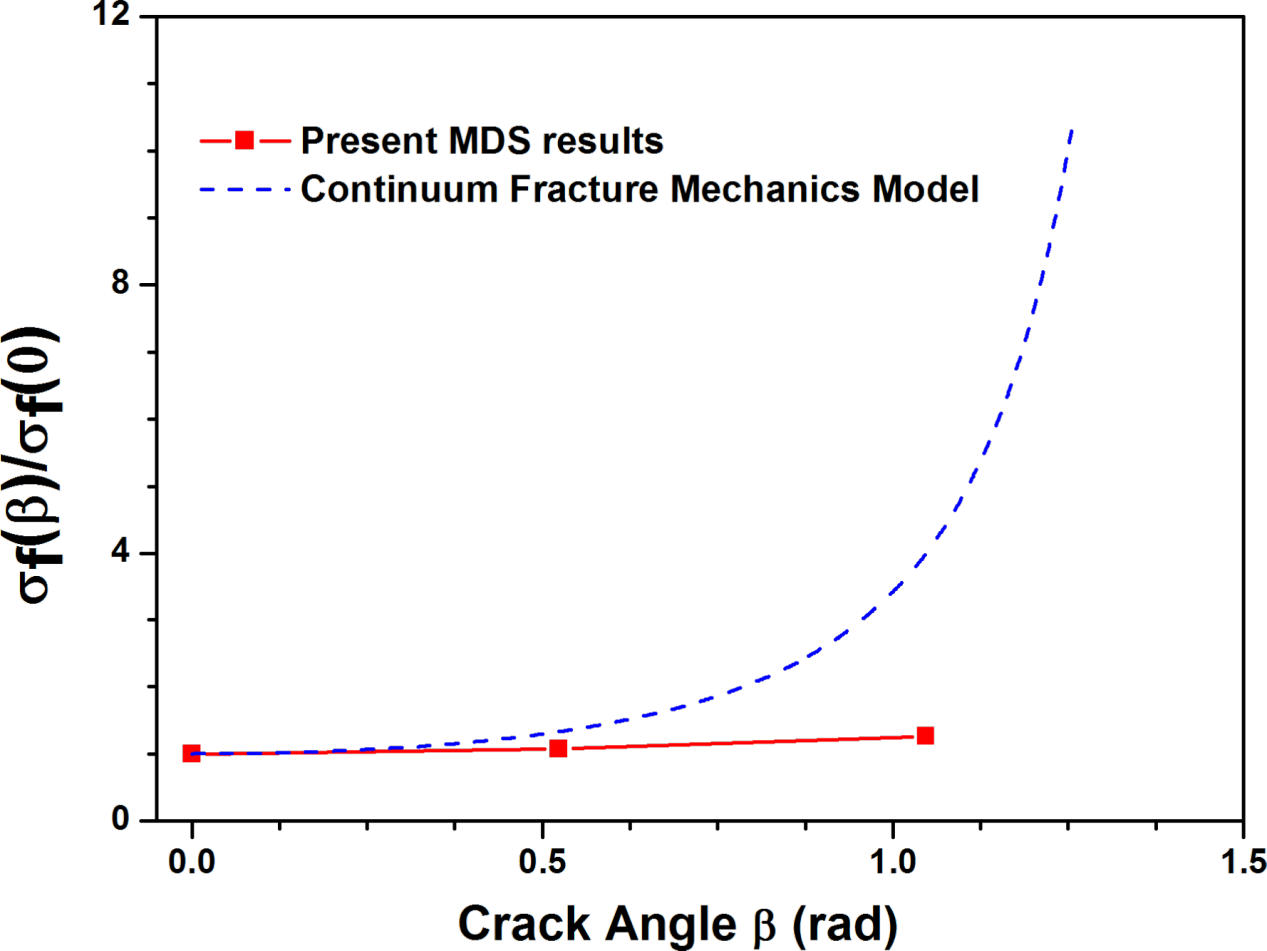
Comparison of fracture strength of MoS_2_ sheet between MDS results and continuum fracture mechanics model versus different crack angles.

**Figure 15:**
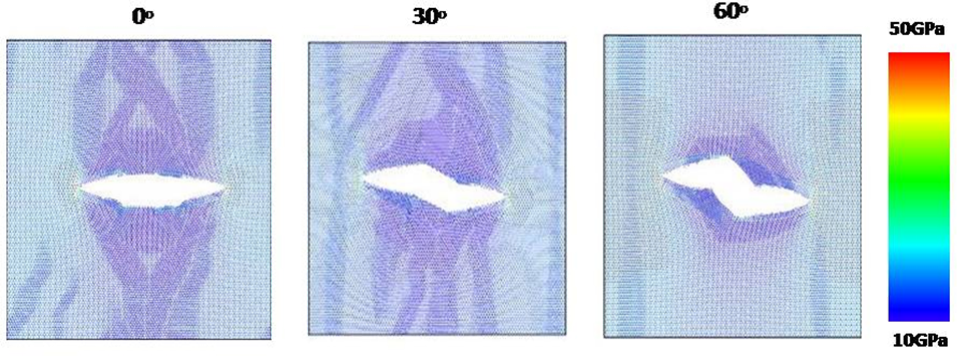
The von Mises stress distribution after crack extension for three crack angles.

Figure 13 shows the stress–strain curves along armchair direction of pre-cracked MoS_2_ sheet of 5 nm length crack with different crack angles. As the crack angle increases, the fracture strength increases gradually. By making comparison of fracture strength of pre-cracked MoS_2_ sheet between MDS results and continuum fracture mechanics model as shown in Figure 14, it is found that there are significant differences between them when the crack angle is over 30 degree. This indicates the continuum fracture mechanics model fails on the prediction of fracture strength of pre-cracked MoS_2_ sheet when the crack angle is over 30 degree. That is to say, only when the crack angle is less than 30 degree, the theoretical prediction is reliable and credible. Figure 15 shows the von Mises stress distribution after crack extension for three crack angles, the crack propagation is along the direction perpendicular to the loading direction even if the crack angle is large (60 degree). This demonstrates that we can introduce the equivalent Mode I crack to replace the angled crack for simplification of the problem when the crack propagation is investigated.

## Conclusion

The fracture strength and crack propagation of pre-cracked MoS_2_ sheets with different cracks are investigated by performing MDS. The results are summarized as follows:

1. The crack tip configuration can significantly influence the fracture strength and crack propagation of pre-cracked MoS_2_ sheets. With the increase of kink angle of crack tip, the fracture strength increases gradually. And the atomic bond formed at the crack tip is stronger, the fracture strength is higher. Additionally, the crack tends to propagate along the kink angle of crack tip.
2. For the crack with equal length, as the model size increases, the fracture strength increases slightly and the fracture is gradually transformed from brittle to ductile. The location of crack does not markedly influence the fracture behaviors of pre-cracked MoS_2_ sheets.
3. With the increase of crack length, the fracture strength of pre-cracked MoS_2_ sheet reduces almost bi-linearly and the results agree well with the theoretical prediction of the Griffith's model with the finite size effect be considered. The Inglis' model and Griffith's model without considering finite size effect cannot predict the results accurately.
4. For the angled crack, the continuum fracture mechanics model fails on the theoretical prediction of fracture strength of pre-cracked MoS_2_ sheet when the crack angle is beyond 30 degree. However, the equivalent Mode I crack model can be used to simplify the problem of angled crack when the crack propagation is of interest.

## Supporting Information

Supplementary data associated with this article is the animation on the structure transition of MoS_2_ sheet under tension. From animation, the structure consisting of molybdenum and sulfur atoms varies suddenly at the engineering strain of about 18%.

File 1 View 1 of structure transition of MoS_2_.

File 2 View 2 of structure transition of MoS_2_.
